# Sexual Quality of Life-Female (SQoL-F): Translation, cultural adaptation and validation of the Brazilian Portuguese version in postpartum women

**DOI:** 10.61622/rbgo/2025rbgo32

**Published:** 2025-07-15

**Authors:** Dulciane Martins Vasconcelos Barbosa, Denise Nicodemo, Edmércia Holanda Moura, Silvania de Cassia Vieira Archangelo, Lydia Masako Ferreira, Daniela Francescato Veiga

**Affiliations:** 1 Universidade Federal de São Paulo São Paulo SP Brazil Universidade Federal de São Paulo, São Paulo, SP, Brazil.; 2 Centro Universitário Facid Wyden Teresina PI Brazil Centro Universitário Facid Wyden, Teresina-PI, Brazil.; 3 Universidade do Vale do Sapucaí Pouso Alegre MG Brazil Universidade do Vale do Sapucaí, Pouso Alegre, MG, Brazil.

**Keywords:** Sexuality, Quality of Life, Postpartum period, Surveys and questionnaires

## Abstract

**Objective:**

Sexuality plays an important role in quality of life, and the postpartum period may negatively affect women's sexual function. This study aimed to translate, culturally adapt, and validate the Sexual Quality of Life – Female (SQoL-F) for use for use in Brazilian women in the postpartum period.

**Methods:**

The original version of the SQoL-F was translated and back translated by four independent sworn translators. A sample of 125 women in the late postpartum phase participated in the cultural adaptation (n=30) and convergent validation (n=95) phases. For the latter, the instrument was compared with the Brazilian version of the Female Sexual Function Index (FSFI). To assess reproducibility, 25 of the 95 women who participated in the validation phase completed the SQoL-F twice, at different times (two interviewers administered the SQoL-F, 15 to 20 days apart).

**Results:**

Cronbach's alpha was 0.905 (intraclass correlation=0.974; 95%CI: 0.943–0.988; p<0.001). Significant, moderate, positive correlations were observed between the SQoL-F score and the FSFI total score (r=0.572; p<0.001) and domains ‘Desire’ (r=0.502; p<0.001), ‘Arousal’ (r =0.576; p<0.001), and ‘Satisfaction’ (r=0.637; p<0.001). Excellent reproducibility was obtained for the SQoL-F score (intraclass correlation=0.974; 95%; CI: 0.943–0.988; p<0.001).

**Conclusion:**

The SQoL-F was adapted to the cultural context of Brazilian postpartum women, proved reproducible, and exhibited face, content, and construct validity.

## Introduction

The human sexual response is the result of interactions between biological and psychosocial factors, which vary between cultures and individuals.^(1,2)^ Sexuality is a key dimension of the QoL of individuals, families, and communities.^(1)^ In Brazil, a study with 2,835 participants found that complaints related to sexual disorders were highly prevalent. In women, the most common dysfunction reported was a lack of sexual desire.^(3)^ Female sexual dysfunction (FSD) has a multifactorial etiology and can be triggered by trauma, hormonal changes, menopause, childbirth, and breastfeeding, among other events.^(2,4)^

Sexual function decreases significantly after childbirth, and negative body image can affect a woman's sex life during this period. Cultural factors can also influence postpartum sexual behavior, as some cultures frown upon or even outright forbid sexual intercourse during this period.^(5,6)^

Challenges in identifying and treating FSD include women's discomfort regarding the topic, a reproduction-focused culture, and inadequate training of healthcare providers to address this issue.^(2)^ Women are inhibited from talking about sex, which may be one of the causes of underreporting of FSD to healthcare providers.^(3)^ As a result, FSD is both underestimated and insufficiently studied.^(2,6)^

One of the instruments employed most widely to assess sexual function is the Female Sexual Function Index (FSFI), which has been validated for use in Brazil.^(7-10)^ Although it is a reliable instrument, it only assesses physical aspects of sexual function and does not cover any sexual-related quality of life (QoL) dimensions.^(9)^

Impairment of sexual function can lead to worsening of general well-being and overall QoL.^(7,11-13)^ The Sexual Quality of Life – Female (SQoL-F) instrument was designed to supplement assessment of the physical aspects of sexual functioning, encompassing aspects related to women's QoL. Its items reflect three specific areas of impact of FSD: self-esteem, emotional issues, and relationship issues.^(14)^

Considering the importance of identifying FSD in the postpartum phase and aiming to better understand sexual function and its impact on women's QoL during this period, we aimed to translate the SQoL-F into Brazilian Portuguese, adapt it to the cultural context of postpartum women, and validate it for use in Brazil.

## Methods

First, we obtained authorization from the copyright holders of the instrument to carry out the adaptation process. The sample size was calculated by multiplying the number of items in the instrument (18) by 5, which resulted in a minimum sample of 90 participants for validation.^(13)^ An additional 30 women were selected for the cross-cultural adaptation phase.^(6)^ To account for possible losses, a total of 125 women in the late postpartum period (between 45 and 180 days postpartum), between the ages of 18 and 40 years, were included. All were recruited from public healthcare facilities in the city of Teresina, Piauí, Brazil. Data collection was carried out between March 2022 and June 2023.

The SQoL-F is a self-report instrument consisting of 18 items scored on a Likert-type scale from 1 (completely agree) to 6 (completely disagree). Items 1, 5, 9, 13 and 18 are reverse-scored and must be converted before calculating the sum score. The total score ranges from 18 to 108. Higher scores denote a better sexual quality of life.

The translation process was carried out following international guidelines.^(15-17)^ The original version of the SQoL-F was translated from English into Brazilian Portuguese by two independent sworn translators. Only one of them was aware of the objective of the study.

The two translated versions were evaluated and compared by a multidisciplinary, bilingual group, composed of a nurse, a plastic surgeon, a psychologist, and a gynecologist, who considered idiomatic, semantic, conceptual, and cultural equivalences. This group produced a consensus version of the instrument, in Portuguese, which was then back translated into English by two other independent sworn translators, neither of whom was aware of the objectives of the study.

The two back-translated versions were analyzed and compared to the original scale by the same multidisciplinary group, which then constructed a new consensus version keeping the essential characteristics of the original scale in English. A report of this process was sent to the authors of the original instrument. Upon their approval, the consensus Brazilian Portuguese version moved on to the next stage: adaptation to the linguistic and cultural context of the target population.

To adapt it to the target linguistic and cultural context, the SQoL-F was administered to 30 women from the target population. The items were well understood by the participants. Only two stated they did not understand the word "frustrated" in item 11, which was then reviewed by the multidisciplinary group to establish the final version of the instrument.

Psychometric properties were evaluated by assessment of face and content validity, as determined by the relevance of each item, and based on the consensus opinion of the multidisciplinary team.

Reliability tests were carried out based on the determination of internal consistency (of the scale as a whole and by factors) and assessment of inter- and intra-rater reproducibility. To assess reproducibility, a test-retest method was applied: 25 women from the target population were selected and completed the SQoL-F twice, at different times (two interviewers administered the SQoL-F, 15 to 20 days apart).

Construct validity was verified by evaluating divergent validity (differences between aspects of satisfaction based on participants’ characteristics), factor analysis (analysis of dimensionality of the latent construct), and convergent validity. For assessment of convergent validity, 95 women from the target population were selected and completed both the SQoL-F and the FSFI.^(9)^

To evaluate the dimensionality of the scale, confirmatory factor analysis (CFA) and exploratory factor analysis (EFA) were employed, using the principal components method and varimax orthogonal rotation. The internal consistency of the scale (overall and among items in each domain) was analyzed using Cronbach's alpha coefficient.

Convergent validation was observed based on linear associations between SQoL-F and FSFI scores, as evaluated via Spearman correlation, due to the non-normal distribution of some scores. The Kolmogorov–Smirnov test was used to assess the normality of data distribution. The reproducibility of the SQoL-F total and domain scores was evaluated via intraclass correlation. For all tests, statistical significance was accepted at the 5% level. All analyses were carried out in SPSS Version 20.0 and STATA 17.

This project was approved by the institutional research ethics committee 4.070.422 (*Certificado de Apresentação de Apreciação Ética:* 29768020.0.0000.5505) and was conducted in strict accordance with the tenets of the Declaration of Helsinki. All participants provided written informed consent.

## Results

The profile of the 125 women who participated in the study is described in table 1.

**Table 1:** Descriptive characteristics of the respondents

Age (years)		n(%)
Mean ± SD		27.9 ± 6.1
Educational attainment		
	Primary education	42(33.6)
	Secondary education	61(48.8)
	Higher education	22(17.6)
Obstetric history		
	Primigravid	53(42.4)
	Multigravid	72(57.6)
Mode of delivery		
	Normal	43(34.4)
	Cesarean	82(65.6)
Cohabitates with partner		
	Yes	76(80.0)
	No	19(20.0)
Timing of first postpartum sexual activity		
	≤30 days postpartum	6(4.8)
	30 to 40 days postpartum	30(24.0)
	40 to 50 days postpartum	48(38.4)
	>50 days postpartum	41(32.8)
Sexual dysfunction reported to a healthcare provider		
	Yes	9(7.2)
	No	116(92.8)

SD: standard deviation

**Figure 1:**
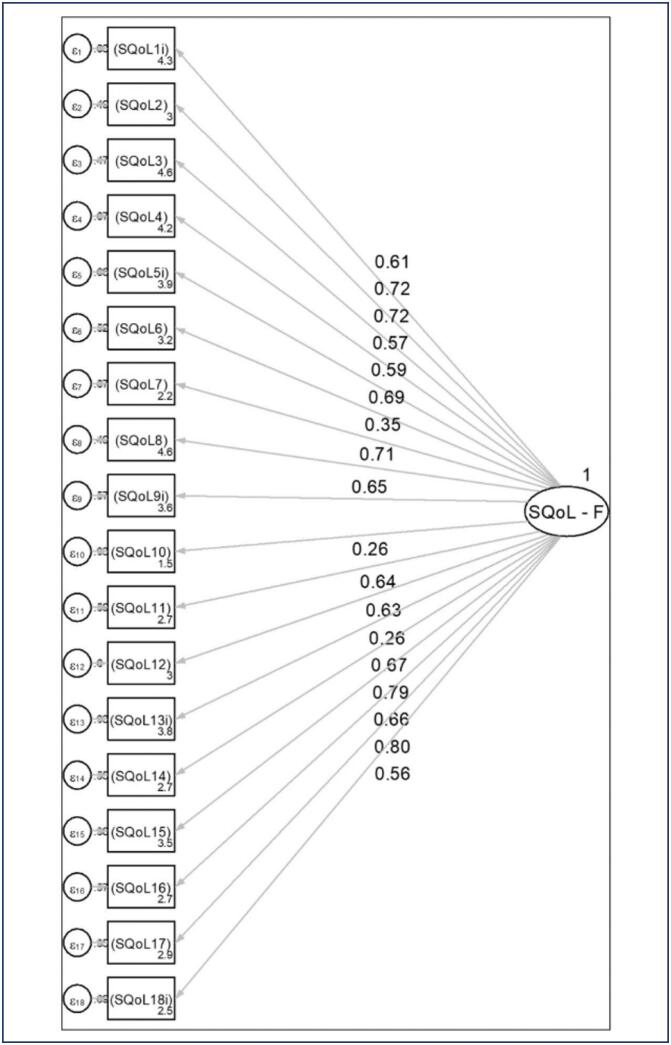
Estimates of the confirmatory factor analysis model for the SQoL-F

They all declared themselves heterosexual. The time taken to complete the questionnaire ranged from 5 to 8 minutes. Six items of the SQoL-F presented a high concentration (at least 34%) of "completely agree" answers, suggesting the presence of a floor effect. Furthermore, 12 items had at least 29% "completely disagree" responses, indicating a ceiling effect. The SQoL-F scale consists of 18 items and has a single dimension. CFA was used to assess the plausibility of this model. The items were found to correlate moderately/strongly and significantly with the factor in which they are included, except for items 7 (" When I think about my sexual life, I feel anxious"), 10 (" I worry about the future of my sexual life"), and 13 (reverse-scored) (" When I think about my sexual life, I feel that I can talk to my partner about sexual matters"), which showed significant but weak correlations (ranging from 0.259 to 0.354) (Table 2; Figure 1).

**Table 2:** Estimated item–factor correlations

SQoL-F Items	Coefficient (CFA)	95%CI	SE	p-value
1.	0.609	0.492 - 0.727	0.060	<0.001
2.	0.716	0.622 - 0.810	0.048	<0.001
3.	0.725	0.632 - 0.817	0.047	<0.001
4.	0.573	0.448 - 0.699	0.064	<0.001
5.	0.587	0.465 - 0.710	0.062	<0.001
6.	0.690	0.590 - 0.789	0.051	<0.001
7.	0.354	0.193 - 0.514	0.082	<0.001
8.	0.714	0.620 - 0.807	0.048	<0.001
9.	0.654	0.545 - 0.763	0.056	<0.001
10.	0.259	0.088 - 0.430	0.087	<0.001
11.	0.642	0.532 - 0.753	0.056	<0.001
12.	0.632	0.519 - 0.745	0.058	<0.001
13.	0.260	0.089 - 0.431	0.087	<0.001
14.	0.674	0.571 - 0.777	0.053	<0.001
15.	0.789	0.714 - 0.864	0.038	<0.001
16.	0.656	0.548 - 0.764	0.055	<0.001
17.	0.803	0.733 - 0.874	0.036	<0.001
18.	0.559	0.431 - 0.687	0.065	<0.001

CFA: Confirmatory Factor Analysis; 95%CI: 95% confidence interval; SE: standard error; p: descriptive level of significance (CFA)

EFA was then carried out to evaluate the dimensionality suggested by the data. This analysis identified four factors (four eigenvalues greater than 1) that explained 63.66% of the total variance of the 18 items. However, these four factors did not allow a clear interpretation considering the correlations between the factors and the items. Therefore, we decided to keep the original, unidimensional SQoL-F model comprising all 18 items.

Excellent internal consistency (Cronbach's alpha = 0.905) was observed among the items. The correlations between items 7, 10, and 13 and the score composed of all items on the scale (item–total correlation), excluding each of the items in question, were low (less than 0.40), confirming the results of CFA. Exclusion of item 10 resulted in an increase in Cronbach's alpha coefficient to 0.912. Similar results were obtained for items 7 and 13.

**Figure 2:**
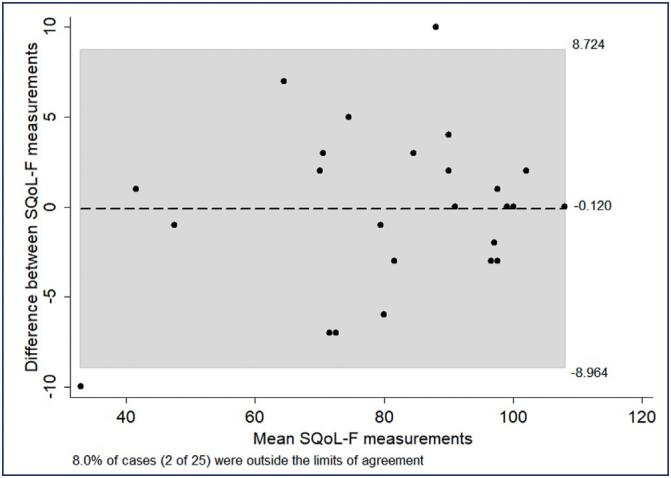
Bland-Altman plot for the SQoL-F total score

For assessment of reproducibility, data from 25 women were considered. Excellent reproducibility was obtained for the SQoL-F score (intraclass correlation=0.974; 95%CI: 0.943–0.988; p<0.001); fewer than 10% of cases were outside the limits of the confidence interval. However, greater dispersion was observed for lower scores (Figure 2).

Data from 95 women were used for assessment of convergent validity. Significant, moderate, positive correlations were observed between the SQoL-F score and the FSFI total score (r=0.572; p<0.001) and with domains ‘Desire’ (r=0.502; p<0.001), ‘Arousal’ (r =0.576; p<0.001), and ‘Satisfaction’ (r=0.637; p<0.001). Correlations between the SQoL-F score and the FSFI domains ‘Lubrication’ (r=0.389; p<0.001), ‘Orgasm’ (r=0.374; p<0.001), and ‘Pain’ (r=0.217; p=0.035) were significant, but weak.

## Discussion

This study applied standardized methods to translate and validate the SQoL-F for use in Brazilian postpartum women. The SQoL-F was successfully adapted to the cultural context of Brazilian postpartum women, proved reproducible, and exhibited face, content, and construct validity. The availability of a validated, practical and reliable self-report instrument has the potential to help healthcare providers to understand sexual function and its impact on Brazilian women's quality of life during the postpartum period.

A study of 100 Brazilian women found a significant prevalence of sexual issues and dysfunctions, demonstrating the importance of sexuality education work and clinical interventions for improving sexual quality of life, particularly in public health.^(18)^ In this context, it is essential to assess the status of women's sexual health, just as it is important to provide healthcare workers with simple, effective tools to assess female sexual health.

A systematic review demonstrated that the FSFI is one of the most used tool to assess female sexual function in Brazil, and the one which was most validated for different clinical populations.^(19)^ Recently, a specific version for women with breast cancer has been translated, culturally adapted and validated for use in Brazil.^(20)^ Further, the Brazilian Portuguese version of the Female Sexual Function Index 6-item (FSFI-6) was tested for internal consistency, reliability, and criterion validity in postpartum women, proving to be valid for use in this population.^(21)^

The psychometric tests carried out in the present study demonstrated that the SQoL-F is a valid, reliable instrument for assessing sexual quality of life in postpartum Brazilian women. The SQoL-F has already been used by other authors to assess the sexual QoL of women with adenomyosis, acne, status post myocardial infarction, high-risk pregnancy, infertility, pregnancy and postpartum.^(22-27)^

Pereira et al.^(28)^ recently published a translation and adaptation of the SQoL-F into Brazilian Portuguese. However, important differences exist in relation to the present study: their translation process was different, the instrument was administered electronically, and their sample covered the general population. In our study, we chose to include postpartum women as the target population, considering this is a particularly delicate period in a woman's life, beset with physical, psychological, and sociocultural changes that can potentially affect sexual function.^(2,4)^ Furthermore, a literature search yielded no studies that explored sexual QoL using a valid, reliable instrument in Brazilian women in the postpartum period.

Like previous work, the present study included only women of reproductive age.^(12,28)^ The authors of the original version of the instrument, however, included women aged 19 to 65 years (thus encompassing the premenopausal and menopausal periods), which limits comparisons of our findings to theirs.^(14)^

Regarding the dimensionality of the scale, the SQoL-F items reflect three specific areas of potential impact on sexuality: self-esteem, emotional issues, and relationship issues. However, they yield a single score, which does not allow evaluation of separate domains. Indeed, the confirmatory factor analysis carried out in the present study demonstrated weak correlation for three items on the scale (7, 10, and 13). To evaluate the dimensionality suggested by this result, an exploratory factor analysis was carried out, which suggested the existence of four underlying factors. However, these four factors did not allow a clear interpretation considering the correlations between the factors and the items. Therefore, we decided to keep the unidimensional model for the Brazilian version of the SQoL-F.

Maasoumi et al.^(12)^ obtained similar results when evaluating the psychometric properties of the Iranian version of the SQoL-F, also uncovering four factors: (1) psychosexual feelings; (2) sexual and relationship satisfaction; (3) self-worthlessness (a construct evaluating negative feelings of oneself); and (4) sexual repression. The authors argued that this finding could be related to sociocultural differences, which may lead women to respond differently to sexuality-related issues.^(12)^

Pereira et al.^(28)^ found a two-factor solution for their version of the SQoL-F; however, analysis of the factor loadings of the rotated items did not provide a good rationale. Since most of the items exhibited cross-loadings, the unidimensionality of the scale was preserved. Therefore, future investigations to confirm the dimensionality of the SQoL-F are warranted.

a valid and reliable questionnaire is required. The aim of this study was to translate and validate the Sexual Quality of Life-Female (SQOL-FThe internal consistency of the instrument was excellent (α=0.905), similar to that observed by Pereira et al.^(28)^ (α=0.950) and higher than those obtained for the Iranian version (α=0.730) and for a European Portuguese version (α=0.800).^(12,28,29)^ Test-retest reliability was also confirmed, based on the observation of excellent reproducibility for the SQoL-F score. Although greater dispersion was observed in lower scores, this finding can be justified by fluctuations in the perceptions of women who have poor sexual QoL.

The European Portuguese version of the SQoL-F demonstrated poor convergent validity when compared to the Questionnaire on Attitudes and Beliefs about Sexuality and Sexuality Education (QABSSE or ABQSSE); this was justified by the authors by the fact that the latter is a multidimensional instrument.^(29,30)^ In the present study, convergent validity – assessed by comparison of SQoL-F and FSFI scores – was demonstrated by significant moderate and positive correlations. Higher SQoL-F scores (indicating better sexual quality of life) were associated with higher FSFI total scores and Desire, Arousal, and Satisfaction domain scores.^(9)^ This confirms the reliability of the instrument to assess sexual function as a whole, including physical aspects and those related to the different dimensions of QoL.

It is worth highlighting the difficulty of finding participants with specific eligibility criteria, in a sensitive period as the postpartum period, willing to make their time available for interviews about sexual function, especially considering that data collection was carried out shortly after the COVID-19 pandemic. The application of the instrument through a face-to-face interview, despite presenting logistical challenges, provides greater reliability in the responses, especially in comparison with studies that use an online approach.

Limitations of the present study include a non-probabilistic sampling and the single-center design, with patients recruited from a public health clinic in a single region, which could limit its external validity and applicability. Furthermore, we did not include a control group of non-postpartum women.

However, the availability of the SQoL-F for use in Brazil will allow studies to be carried out that should contribute significantly to clinical practice and add to the body of evidence into the sexual quality of life of women in the postpartum period.

## Conclusion

The SQoL-F was adapted to the cultural context of Brazilian postpartum women, proved reproducible, and exhibited face, content, and construct validity.
